# Supplementary material for: Experiences of Caregivers and At-Risk Children Enrolled in a Prospective Pregnancy-Birth Cohort Study into the Causes of Type 1 Diabetes: The ENDIA Study
Source: Children (Basel). 2023 Mar 29;10(4):637. doi: 10.3390/children10040637 (PMC10137087; doi:10.3390/children10040637)
Supplement: Supplementary file 1 [file children-10-00637-s001.zip › 221209_ENDIA Supplementary Information 1.pdf]

ENDIA Participant Experience Survey

Participant ID: [redacted]

Thank you for participating in the ENDIA Study. We value all the information you have provided to us to help find the causes of type 1 diabetes. It is important for us to learn about your experience of this research; what is working well for you and ways that we can improve.

To help us evaluate if we provide an appropriate child and family-centred research experience, we ask your child to complete the Faces Scale Questionnaire (if they are aged 3 years or over). They may need your assistance to understand the questions being asked of them.

We would also appreciate your perspective by asking you to complete our evaluation questions. This should take no more than 10 minutes.

All survey responses will be sent directly to our Engagement Coordinator in Adelaide, [redacted], for data entry (if not entered directly through the electronic link). Responses will be de-identified and summarised. No specific or identifying information will be shared with other ENDIA staff unless you specify a wish for it to be.

Participant IDs are included so that we can reduce the number of questions in the survey by using data that we already have. Both positive and negative feedback will help improve how we deliver the ENDIA Study. So please do be honest. Information provided, or the decision not to complete this survey, will not affect your ongoing participation or treatment in the ENDIA Study.

We would appreciate you completing this before or during your next visit. You can give your completed questionnaire to your coordinator, you can mail it to [redacted] in the supplied reply-paid envelope, you can scan and email it to [redacted], or you can complete it online at this link: [redacted] If you have already completed this questionnaire, you do not have to complete it again.

We hope this evaluation will help us make participating in the ENDIA Study as positive a research experience as it can be. What we learn might help us to improve future ENDIA visits and how we conduct the Study. Please ask a member of the research team if you have any further questions, or contact our Engagement Officer, [redacted].

This study has been reviewed and approved by the Women's and Children's Health Network Human Research Ethics Committee. Should you wish to discuss matters concerning policies, obtain information about the conduct of the study or your rights as a participant, or should you wish to make a confidential complaint, you may contact the Executive Officer of the committee on [redacted] or email [redacted]

By completing and sending us the answers to this survey you agree that you:

- *have read the above Information, or someone has read it to you in a language that you understand.*
- *understand the purposes, procedures and risks of the research described in the project.*
- *have had an opportunity to ask questions and are satisfied with the answers received.*
- *freely agree to participate in this research project as described and understand that you am free to withdraw at any time during the project without affecting my future health care.*
- *understand that my child's information will be kept confidential except where there is a requirement by law for it to be divulged.*

Thank you so much for your time!

1. What is the date today: / /

2. What is your relationship to the ENDIA child? Tick the box that is right for you:

Mother ☐ Grandparent ☐

Father ☐ Other, please specify ☐

____

3. Did the information and discussions you had at the time of enrolment to ENDIA prepare you for your experience in the study?

No ☐ Yes, mostly ☐

Yes, somewhat ☐ Yes, completely ☐

4. Has anything surprised you whilst participating in the ENDIA Study?

No ☐ Yes ☐ please tell us what:

____

____

____

5. Please tick one box to rate your overall experience in ENDIA:

**1. Terrible**		**2. Bad**		**3. Average**		**4. Good**		**5. Excellent**	
Can you tell us why you gave this rating?									

6. Listed below are some reasons that people might stay in ENDIA. We would like to know how important each of these reasons is to you. Tick the box that is best for you.

Reasons for staying in ENDIA	How important have these reasons been for you?		
	Very Important	Important	Not Important
Knowing someone is monitoring my child for the development of type 1 diabetes			
Knowing someone is monitoring my child for the development of coeliac disease			
To learn more about type 1 diabetes			
To help discover the causes of type 1 diabetes			
Getting my child's diabetes marker results (islet autoantibodies)			
Getting my child's coeliac antibody results			
Getting my child's vitamin D levels			
Positive experience in previous research			
Whole of family involvement			
Knowing my child could participate in future prevention or intervention studies			
Opportunity to screen my other child(ren) for autoantibodies			
Seeing the same ENDIA staff at each visit			
Support and reassurance from ENDIA staff			
To prevent type 1 diabetes for my child			
Study updates and research findings			
Feel valued as a research participant			
Other, please tell us:			

7. Could we do anything to make ENDIA a better experience for you and your family?

____

8. Below is a list of different parts of participating in ENDIA. Please tick the box that best describes how important these parts of the study have been for your experience in ENDIA.

Different parts of ENDIA	Very Important	Important	Not important	Not applicable
Reminders for ENDIA visits				
Parking for ENDIA visits				
Clinic setting or environment				
Home visits				
Day or time ENDIA visits are scheduled				
The length of time an ENDIA visit takes				
How ENDIA staff interact with my child				
How ENDIA staff interact with me				
Able to contact ENDIA staff when I need to				
Gifts for my child (bubbles, slinky, ball, etc)				
Study updates (newsletters, Facebook, etc)				
Timeliness of results				
The way my child's results are given to me				
Other, please tell us:				

9. For the list below, please tick the box that best describes how satisfied you are with these parts of the ENDIA Study.

Different parts of ENDIA	Very Satisfied	Satisfied	Not satisfied	Not applicable
Blood tests				
Collection of swabs				
Collection of urine and stool samples				
Completion of questionnaires				
Using the ENDIA app				
Food intake recall surveys with dietitians				
Receipt of vouchers for visits attended				
Referral to other health professionals				
**Those enrolled in the Regional Participation Program (RPP)**				
Self-collection of samples				
Use and storage of the bio bottle				
Communication with the ENDIA nurse				
Courier collection of samples				
Other, please tell us:				

10. The COVID-19 pandemic changed the way we conducted ENDIA. What impacted you and your family most?

**ENDIA**	**Big impact**	**Some impact**	**No impact**	**Not applicable**
Self-collection of samples				
Storage of samples in the freezer				
No blood test or results for antibodies				
No face-to-face contact with the ENDIA nurse				
**COVID-19**	**Big impact**	**Some impact**	**No impact**	**Not applicable**
Social distancing				
Home life				
Work life				
Child(ren)'s schooling				
Other (please tell us):				

11. Have you ever thought about leaving ENDIA? No ☐ Yes ☐ If so, tick the box(es) that apply:

**Reasons you thought of leaving ENDIA**	**Please tick all that apply**	**Reasons you thought of leaving ENDIA**	**Please tick all that apply**
Blood tests		Time commitment required	
Collection of other samples		Scheduling around work commitments	
Reminder of type 1 diabetes risk		Moved home	
ENDIA staff		Health issues	
Feedback and information about my family's involvement		Changes in family circumstances	
Food diaries or food recalls		Partner not wanting to participate	
Number and length of questionnaires		Transport or parking issues	
Other, please tell us:			

12. Would you be willing for our Engagement Coordinator to contact you to discuss any of your answers?

No ☐
Yes ☐

Please provide your preferred email or phone contact ______________________________

THANK YOU FOR TAKING THE TIME TO GIVE US YOUR OPINION. WE GREATLY VALUE YOUR PARTICIPATION IN THE ENDIA STUDY.

ENDIA Child Experience Survey **Participant ID** ☐☐☐☐☐☐☐☐☐☐☐☐

Hello! You are in the ENDIA Study about type 1 diabetes. Thank you! This is when you see a nurse at hospital or at home. They weigh you and see how tall you've grown, rub your arms, tongue and nose with long skinny swabs, and sometimes take some blood from your vein. We'd like to ask you some questions about it:

1. *How do you feel about being in the ENDIA Study? Colour in a face, or circle the number best for you.*

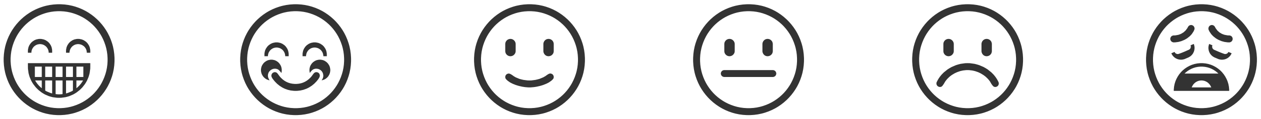

0	2	4	6	8	10
Very happy	Happy	OK	A bit unhappy	Unhappy	Very unhappy

2. *What do you like about being in the ENDIA Study? (use the back of the page if you need)*

____

____

____

3. *What don't you like about being in the ENDIA Study? (use the back of the page if you need)*

____

____

____

Did anyone help you with this question?

Mum	☐	Grandparent	☐
Dad	☐	Other, please tell us:	☐

____

What date is it today? / /
