# Supplementary material for: Experiences of Caregivers and At-Risk Children Enrolled in a Prospective Pregnancy-Birth Cohort Study into the Causes of Type 1 Diabetes: The ENDIA Study
Source: Children (Basel). 2023 Mar 29;10(4):637. doi: 10.3390/children10040637 (PMC10137087; doi:10.3390/children10040637)
Supplement: Supplementary file 1 [file children-10-00637-s001.zip › 221209_ENDIA Supplementary Information 2.pdf]

Experiences of caregivers and children in a prospective pregnancy-birth cohort study

Kelly J. McGorm, James D. Brown, Alison G. Roberts, Susan Greenbank, Daniella Brasacchio, Alyssa C. P. Sawyer, Helena Oakey, Peter G. Colman, Maria E. Craig, Elizabeth A. Davis, Georgia Soldatos, Rebecca L. Thomson, John M. Wentworth, Jennifer J. Couper, Megan A. S. Penno, on behalf of the ENDIA Study Group.

ONLINE SUPPLEMENTAL INFORMATION 2

Positive remarks

"As a mum being type 1, I feel like I am helping my kids by being proactive in finding out more about T1DM and with the hope that I can help all families prevent their children from having T1DM or being super ready and comfortable to treat it! Every T1DM should give ENDIA a go - ENDIA and its participants help each other. It's a positive cycle that never stops giving."

"I found the initial years when the children were young (infants) incredibly time intensive and I don't think I was prepared for this. Saturdays, home visits and the VR goggles all make it easier to be involved. Breaking up the tasks so some can be done pre/post visit also helps."

"Throughout our entire ENDIA experience we have had positive experiences. To the point where these positive experiences have become relevant when getting vaccinations. My children have no fear of needles or medical intervention due to the positive, fun engagement of our ENDIA nurses.".

"I feel very pleased to be contributing to research in this area, and am proud of the commitment required to fulfil the study requirements, particularly in the first year or two of the child's life. I have had pleasant interactions with all the staff, and when my son was diagnosed with an autoimmune condition through his participation in the study, I thought the follow-up arranged was excellent. I feel he has actually benefited from being in the study, which was not something I expected from participating in the research.".

"The staff are lovely and very good with my kids. They were very understanding when my child was very nervous about having a blood test. They talked her through it step by step but didn't push her. She now doesn't mind them at all. The staff have also been wonderful keeping me informed of the blood test results and what they mean. Also the Facebook page/website is really good for research updates/videos etc"

"It's not just collecting data but also bringing together information for me as a type 1 father and connecting me to a community. The health information I've been able to receive as a result of our participation has really saved me."

"Our overall experience of ENDIA has been excellent - it gives us as a family great pride in knowing that we can help by providing invaluable samples to science in a bid to find a cure or at least find the reason that type 1 occurs."

Negative remarks

“My experience was as I expected and my rating of average is not a fault of the research team/nurses. It is worthwhile because the info has been great and the team accommodated additional tests of his blood for me and were all very nice. However, the blood tests were so stressful for me and unpleasant for my son. I do acknowledge that the research team always said they weren't compulsory. The study was great but I did find I was anxious when appointments came up.”

“It became a bit inconsistent & I wasn't hearing whether it was making a difference.”

“Because I was never told about any of the information gathered. I had to ask several times for my daughters blood results. No information was ever offered. I think it's an incredibly important study but communication with participants could have been better. Electronic questionnaires would have been helpful too.”

“How quickly six months rolls around. How each time my child has a different experience. How she now remembers, and the struggle of me balancing my choice to enrol her, and her autonomy to choose whether she participates. The guilt associated with this.”

“Would like updates on study results, no need for plastic toys etc,”

“My child gets excited about these gifts, but I do wonder if you might find some more enviro friendly options with less plastic.”

“I just wish there was a pain free way to take the bloods from babies/small kids as watching him cry is the awful part but I know there's not & it's the only way to help everyone towards a diabetes free future..”

Regional Participation Program remarks

“We live rurally, taking some of the swabs ourselves, data (e.g. food) and arranging couriers has been stressful at times while balancing other things over the day. How the study has been completed however has been great, really appreciate that it is thorough and the staff we've engaged with really care.” Regional mum 1.

“Everyone (my husband, both children in the study, our parents and the rural medical practitioners) have been provided with support, care, compassion and love during the study so far.”
